# Association between blood cadmium levels and the risk of chronic kidney disease in Korea, based on the Korea National Health and Nutrition Examination Survey 2016–2017

**DOI:** 10.2478/abm-2025-0004

**Published:** 2025-02-28

**Authors:** Jieun Yeon, Suji Kang, Jiyeon Park, Jin Hee Ahn, Eun-Ah Cho, Sung Hyun Lee, Kyoung-Ho Ryu, Jae-Geum Shim

**Affiliations:** Department of Anesthesiology and Pain Medicine, Kangbuk Samsung Medical Center, Sungkyunkwan University School of Medicine, Seoul 03181, Republic of Korea

**Keywords:** cadmium, chronic kidney disease, diabetes, hypertension, KNHANES

## Abstract

**Background:**

Exposure to environmental cadmium can have harmful effects on the human kidneys. The relationship between the degree of exposure to cadmium and the risk of chronic kidney disease (CKD) in the general population is unclear.

**Objectives:**

To investigate the association between blood cadmium levels and CKD risk using samples from the Korea National Health and Nutrition Examination Survey (KNHANES) VII data, which included heavy metal and serum creatinine levels.

**Methods:**

We analyzed the data of 4,222 adults from January 1, 2016, to December 31, 2017. Multiple logistic regression analysis was conducted on weighted data using complex sampling to assess the relationship between blood cadmium levels and CKD. We performed a stratified analysis in the presence of comorbidities such as diabetes or hypertension.

**Results:**

There was a positive association between blood cadmium level and the risk of CKD in hypertensive or nondiabetic participants after adjustment, but not between blood cadmium level and CKD in normotensive or diabetic participants. The corresponding odds ratios (OR) of cadmium for CKD were 2.70 (95% confidence interval [CI], 1.49–4.90, *P* = 0.001) in samples with hypertension and 2.40 (95% CI, 1.56–3.70, *P* < 0.001) in samples without diabetes.

**Conclusions:**

Our findings suggest an association between blood cadmium level and the risk of CKD in hypertensive or nondiabetic participants. Additional research is necessary to elucidate the relationship between cadmium exposure and CKD risk, particularly in individuals with comorbidities.

Chronic kidney disease (CKD), characterized by renal dysfunction, is a growing public health problem with a profound impact on individuals and society as a whole [1]. CKD is currently one of the most important causes of death and morbidity [2]. As risk factors such as diabetes and hypertension gradually increase, the prevalence of CKD continues to increase, and as of 2017, >10% of the world's population, that is, 800 million people, are suffering from CKD [2, 3]. Cadmium is one of the environmental heavy metals and is present in the air, soil, seafood, cigarettes, and children's plastic toys [4]. Therefore, humans are commonly exposed to cadmium which gradually accumulates in the body. The kidneys are the main organs affected by environmental cadmium exposure during everyday life and are also vulnerable to cadmium toxicity [5]. However, the association between exposure to cadmium and CKD in the population nationwide is not yet clear.

While acute cadmium toxicity is now rare in most countries, chronic cadmium toxicity is mainly characterized by dysfunction of the kidney's proximal tubule, leading to a decreased glomerular filtration rate (GFR). Although the general effect of cadmium on proximal tubule function is known in respect of large quantities through previous studies, the basic mechanism for this effect is not adequately revealed [6]. Oxidative stress has been reported to cause renal tissue damage and is an important contributor to chronic inflammation leading to further tissue injury [7, 8]. Some researchers have shown that cellular death could also be mediated by lipid peroxidation induced by cadmium [9, 10].

The objective of the present study was to evaluate the hypothesis that people with high blood cadmium levels are at a greater risk of developing CKD compared with people with low blood cadmium levels. We examined this in the Korea National Health and Nutrition Examination Survey (KNHANES) VII (2016–2017). Kidney damage was evaluated by measuring an estimated glomerular filtration rate (eGFR). To control for other risk factors of CKD, we performed a stratified analysis in the context of comorbidities, including diabetes and hypertension.

## Methods

### Study population

**Figure 1.**
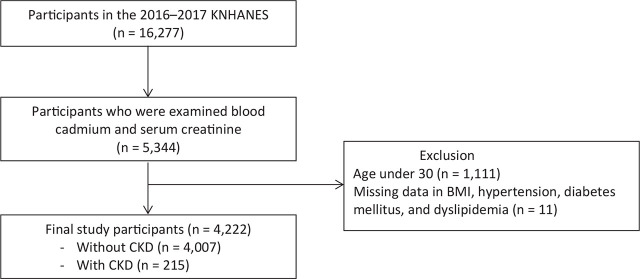
Schematic diagram of participant selection. BMI, body mass index; CKD, chronic kidney disease.

The present study utilized samples from the KNHANES VII (2016–2017) survey data, which included heavy metal and serum creatinine levels. The KNHANES is a nationally representative, population-based, cross-sectional survey conducted each year by the Korea Centers for Disease Control and Prevention (KCDC) [11]. It assessed the health and nutritional status of Koreans, which consisted of 3 components: health interview, health examination, and nutrition. The KNHANES data are publicly available and can be obtained from the KNHANES website (https://knhanes.kdca.go.kr). In the 2016–2017 KNHANES, 16,277 participants were selected by using two-stage stratified cluster sampling. In this study, all populations from the 2016 and 2017 KNHANES with measured blood cadmium levels and serum creatinine concentrations were included. We excluded those under 30 years of age and those with incomplete information such as missing data on body mass index (BMI), hypertension, diabetes mellitus, and dyslipidemia from our analysis (**Figure 1**).

### Blood cadmium levels

The blood cadmium levels were measured partly by randomly subsampling in the KNHANES in 2016 and 2017. The concentration of blood cadmium was assessed by graphite furnace atomic absorption spectrophotometry (GFAAS) using AAnalyst600 (PerkinElmer, Turku, Finland) and presented as microgram per liter.

### CKD

In the current study, CKD was defined as the eGFR <60 mL/min/1.73m^2^ according to the Kidney Disease Improvement Global Outcome (KDIGO) 2012 guidelines [12]. The eGFR was calculated using the following formula according to the Modification of Diet in Renal Disease (MDRD):

$$\begin{array}{c} \text{eGFR}\;(\text{mL}/\min/1.73\;{\text{m}}^{2})=175\times [\text{serum}\;\text{creatinine}\;(\text{mg}/\text{dL})]-\\ 1.154\times (\text{age})-0.203\times 0.742\;(\text{if}\;\text{female}) \end{array}$$

Unfortunately, in the KNHANES VII (2016–2017), the urine albumin-to-creatinine ratio (ACR) value was not available because the urine albumin was not measured.

### Confounders

Covariate variables to minimize confounding bias included age, sex, BMI, and physician-diagnosed hypertension, diabetes mellitus, or dyslipidemia. Age was categorized into 3 groups: 30–50 years, 50–70 years, and >70 years. Hypertension, diabetes, and dyslipidemia were evaluated by the question, “Have you ever been diagnosed with hypertension, diabetes, or dyslipidemia by a physician?”

### Ethical statement

This study protocol was approved by the Institutional Review Board of the Kangbuk Samsung Hospital (2022-07-051). All participants provided written informed consent to the Korea Center for Disease Control and Prevention.

### Statistical analysis

General participant characteristics according to CKD were compared with the Student's *t* test for continuous variables and the chi-squared test for categorical variables. As the KNHANES follows a multistage clustered probability design, the sampling weights for each participant were constructed for unbiasedly representing the Korean population. The weights are adjustment factors assigned to each individual that account for the complex survey design as well as survey nonresponse and post-stratification. Multiple logistic regression analysis was performed on weighted data using complex sampling to evaluate the association between blood cadmium level and CKD. Multivariable model 1 was adjusted for age and sex, and multivariable model 2 was fully adjusted for age, sex, hypertension, diabetes mellitus, and dyslipidemia. The statistical analyses were conducted using SPSS version 24.0 for Windows (IBM, Armonk, NY, USA).

## Results

We used the data of 4,222 adults who participated in the KNHANES VII from January 1, 2016, to December 31, 2017. The baseline characteristics of the participants are shown in **Table 1**. Among the participants, the prevalence of CKD, hypertension, diabetes mellitus, and dyslipidemia was 215 (5.1%), 1,052 (24.9%), 461 (10.9%), and 831 (19.7%), respectively. The mean concentration of blood cadmium was 1.2 μg/L among all participants, with a higher mean blood cadmium in the CKD group than that in the non-CKD group (1.3 μg/L vs 1.1 μg/L, *P* < 0.001). The CKD group was significantly older, had a higher BMI, a higher proportion of men, and a higher prevalence of hypertension, diabetes mellitus, and dyslipidemia than the non-CKD group.

**Table 1.** Characteristics of the 4,222 adults in the KNHANES

**Parameters**	**All (n = 4,222)**	**CKD (n = 215)**	**CKD5**	**Non-CKD (n = 4,007)**	***P***
Age (years)	54 ± 14	69 ± 10	53 ± 14	53 ± 14	<0.001^†^
30–49	1,737 (41.1%)	85 (39.5%)	1 (%)	1,652 (41.2%)	<0.001^†^
50–69	1,773 (42.0%)	9 (4.2%)	3 (%)	1,764 (44.0%)	
>70	712 (16.9%)	121 (56.3%)	1 (%)	591 (14.7%)	
Sex					
Male (%)	1,869 (44.3%)	112 (52.1)	5 (100)	1,757 (43.8%)	0.021^*^
BMI (kg/m^2^)	24.1 ± 3.5	25.0 ± 3.8	26.0 ± 1.9	24.1 ± 3.5	0.001^*^
Hypertension	1,052 (24.9%)	149 (69.3%)	5 (100%)	903 (22.5%)	<0.001^†^
Diabetes	461 (10.9%)	78 (36.3%)	3 (60.0%)	383 (9.6%)	<0.001^†^
Dyslipidemia	831 (19.7%)	71 (33.0%)	3 (60.0%)	760 (19.0%)	<0.001^†^
Smoking	1,700 (40.3%)	94 (43.7%)	4 (80.0%)	1606 (40.1%)	0.29
Blood Cd (μg/L)	1.2 ± 0.6	1.3 ± 0.6	1.4 ± 0.9	1.1 ± 0.6	<0.001^†^

Data show mean ± standard deviation.

* *P* < 0.05.

† *P* < 0.001.

BMI, body mass index; Cd, cadmium; CKD, chronic kidney disease; CKD5, stage 5 of CKD indicating an eGFR of <15 mL/min or less; eGFR, estimate glomerular filtration rate.

In the univariable logistic analysis, we found that the higher concentration of blood cadmium correlated significantly with the prevalence of CKD. After multivariable logistic regression analysis adjusted for age, sex, as well as full adjustment for confounding variables, including hypertension, diabetes mellitus, and dyslipidemia, blood cadmium level had a significant association with CKD, as shown in **Table 2**.

**Table 2.** Multiple logistic regression analysis for CKD by blood Cd in the 4,222 adults in the KHNANES

**Variables**	**OR (95% CI)**	***P***
Univariable model		
Blood Cd	1.59 (1.30–1.94)	<0.001^†^
Multivariable model 1		
Blood Cd	1.34 (1.01–1.79)	0.047^*^
Multivariable model 2		
Blood Cd	2.40 (1.58–3.64)	<0.001^†^

Multivariable model 1 includes adjustment for age and sex. Multivariable model 2 includes adjustment for variables in age, sex, BMI, smoking, dyslipidemia, hypertension, and diabetes.

* *P* < 0.05.

† *P* < 0.001.

BMI, body mass index; Cd, cadmium; CI, confidence interval; CKD, chronic kidney disease.

Additionally, we performed hypertension- and diabetes-stratified analyses of the association between blood cadmium and CKD (**Tables 3 and 4**). There was a positive association between blood cadmium and CKD in hypertensive or nondiabetic participants. However, there was no significant association between blood cadmium and CKD in normotensive or diabetic participants.

**Table 3.** Multiple logistic regression analysis for CKD by blood Cd according to hypertension

**Variables**	**Hypertensive (n = 1,052)**		**Non-hypertensive (n = 3,170)**	

	**OR (95% CI)**	***P***	**OR (95% CI)**	***P***
Univariable model				
Blood Cd	1.39 (1.02–1.91)	0.040^*^	1.72 (1.39–2.14)	<0.001^†^
Multivariable model 1				
Blood Cd	1.51 (1.05–2.15)	0.024^*^	0.94 (0.55–1.62)	0.83
Multivariable model 2				
Blood Cd	2.70 (1.49–4.90)	0.001^†^	2.16 (1.10–4.22)	0.025^*^

Multivariable model 1 includes adjustment for age and sex. Multivariable model 2 includes adjustment for variables in age, sex, BMI, smoking, dyslipidemia, and diabetes.

* *P* < 0.05.

† *P* < 0.01.

BMI, body mass index; Cd, cadmium; CI, confidence interval; CKD, chronic kidney disease; OR, odds ratios.

**Table 4.** Multiple logistic regression analysis for CKD by blood Cd according to diabetes

**Variables**	**Diabetic (n = 461)**		**Nondiabetic (n = 3,761)**	

	**OR (95% CI)**	***P***	**OR (95% CI)**	***P***
Univariable model				
Blood Cd	1.19 (0.72–1.96)	0.500	2.08 (1.69–2.57)	<0.001^†^
Multivariable model 1				
Blood Cd	1.17 (0.68–2.03)	0.569	1.54 (1.08–2.18)	0.016^*^
Multivariable model 2				
Blood Cd	2.32 (0.70–7.75)	0.738	2.40 (1.56–3.70)	<0.001^†^

Multivariable model 1 includes adjustment for age and sex. Multivariable model 2 includes adjustment for variables in age, sex, BMI, smoking, and dyslipidemia.

* *P* < 0.05.

† *P* < 0.001.

BMI, body mass index; Cd, cadmium; CI, confidence interval; CKD, chronic kidney disease; OR, odds ratios.

## Discussion

From the results of analyzing 4,222 adults of KHNANES, there was a significant association between the risk of CKD and age, male sex, BMI, hypertension, diabetes, dyslipidemia, and blood cadmium level. Overall, this reflects consistent results regarding the risk factors of CKD reported in previous studies. However, the blood cadmium levels of all samples in our research were higher than those of previous studies [13, 14]. For instance, in data from the Korean National Human Exposure and Bio-monitoring Examination in 2010, the mean plasma cadmium concentration measured in 2,369 participants was 0.92 μg/L compared with 1.2 μg/L in our study [15]. In another study using data from the Fourth Korea National Health and Nutrition Examination Survey from 2007 to 2008, the mean blood cadmium in a representative sample of 1,709 Korean participants was 1.02 μg/L [16]. It appears that Koreans are gradually becoming more exposed to cadmium. In Korea, smoking, consuming a lot of vegetables, automobile smoke, and fine dust are considered to be sources of cadmium exposure.

Cadmium, a common heavy metal present in the everyday environment, is well known to be introduced into the human body through occupational or environmental exposure, causing adverse health effects [17]. Smoking is known to be a major source of non-occupational cadmium exposure, so the blood cadmium levels in smokers are approximately 3–5 times higher than those in non-smokers [18, 19]. Tobacco contains various metal impurities such as cadmium and lead, with cadmium in particular known to significantly contribute to its accumulation within the body [20]. The influence of smoking and alcohol consumption on blood lead and cadmium levels depends on complex factors ranging from direct exposure increase to absorption and excretion [21]. Severe impairment of renal functions was also a risk factor for high blood cadmium levels, since cadmium is mainly excreted by the kidneys [22]. In this study, 40.3% of CKD patients were smokers, while 43.7% of the non-CKD group were smokers, with no statistically significant difference observed between the two groups. However, given that smoking may have a synergistic effect when accompanied by other risk factors, including alcohol consumption and cardiovascular diseases, even a slight increase of blood cadmium levels should not be disregarded from a public health perspective.

To date, the relationship between the degree of exposure to cadmium and the risk of CKD in the general population is still unclear. In a previous study, Kim et al. [23] reported that cadmium concentration and CKD occurrence correlated in patients with diabetes but not in patients without diabetes. According to the authors, cadmium contributed to the development of CKD with a synergic effect on kidney damage by diabetes and hypertension. However, blood cadmium concentration was associated with a higher risk of CKD in healthy people without diabetes in this study, whereas in patients with diabetes, blood cadmium concentration was not associated with the risk of CKD. Recently, Wang et al. [24] reported that plasma cadmium was negatively associated with CKD in diabetes, which is consistent with our findings. Byber et al. [25] also reported that there was no evidence that exposure to cadmium was related to the risk of progression to CKD. There is still a lack of research on the relationship between low-level environmental exposure to cadmium and the risk of CKD in patients with comorbidities such as diabetes or hypertension. In the future, studies are needed in consideration of Asian genetic characteristics, dietary habits, and lifestyle.

There are limitations to this study. First, since the KNHANES is cross-sectional data, the causality between blood cadmium level and the risk of CKD cannot be determined. Prospective studies are needed to analyze the cause and effect, because only the association at one point can be known. Second, the results of this study were interpreted considering only age, sex, BMI, diabetes, hypertension, and dyslipidemia as the confounding factors. However, many other clinical factors actually should be considered as risk factors for CKD. Researchers have recognized that risk factors for CKD include an individual's genetic factors, race, exposure to heavy metals, excessive alcohol consumption, smoking, and the use of analgesic medications [26]. A history of acute kidney injury, cardiovascular disease, metabolic syndrome, hepatitis C virus, HIV infection, and malignancy are considered to be further risk factors [27]. There is, therefore, a limitation in that the results of this study were derived without fully adjusting for all confounding factors.

The strength of this study is that the effect of blood cadmium level on CKD was analyzed from well-organized, high-quality data called KNHANES. The KNHANES is a national surveillance system that has been evaluating the health and nutritional status of Koreans conducted by the KCDC [11]. KNHANES data are important resources for tracking variations in risk factors and disease trends. The KNHANES is a well-organized survey with nationally representative samples of Korea. Even if our findings are not in part consistent with existing studies, they can be a precursor to studying the potential effects and the underlying mechanism of cadmium.

As a result, even exposure to low-dose environmental cadmium can damage the kidneys and increase the risk of CKD. According to stratified analysis, cadmium concentration was associated with CKD in nondiabetic and hypertensive patients but not in diabetic patients. There might be other reasons that are not clearly identified in diabetic patients, which need to be further investigated.

## References

[1] Dorgelo A, Oostrom TAJ (2022). An integrated approach towards a public health perspective on chronic kidney disease. Nat Rev Nephrol.

[2] Kovesdy CP (2022). Epidemiology of chronic kidney disease: an update 2022. Kidney Int Suppl.

[3] Wang YN, Ma SX, Chen YY, Chen L, Liu BL, Liu QQ (2019). Chronic kidney disease: biomarker diagnosis to therapeutic targets. Clin Chim Acta.

[4] Yan LJ, Allen DC (2021). Cadmium-induced kidney injury: oxidative damage as a unifying mechanism. Biomolecules.

[5] Johri N, Jacquillet G, Unwin R (2010). Heavy metal poisoning: the effects of cadmium on the kidney. Biometals.

[6] Prozialeck WC, Edwards JR (2012). Mechanisms of cadmium-induced proximal tubule injury: new insights with implications for biomonitoring and therapeutic interventions. J Pharmacol Exp Ther.

[7] Lash LH (2021). Diverse roles of mitochondria in renal injury from environmental toxicants and therapeutic drugs. Int J Mol Sci.

[8] Verma S, Singh P, Khurana S, Ganguly NK, Kukreti R, Saso L (2021). Implications of oxidative stress in chronic kidney disease: a review on current concepts and therapies. Kidney Res Clin Pract.

[9] Dong A, Huo J, Yan J, Dong A, Liu B (2021). Lipid peroxidation of kidney of the turtle *Mauremys reevesii* caused by cadmium. Environ Sci Pollut Res Int.

[10] López E, Arce C, Oset-Gasque MJ, Cañadas S, González MP (2006). Cadmium induces reactive oxygen species generation and lipid peroxidation in cortical neurons in culture. Free Radic Biol Med.

[11] Kweon S, Kim Y, Jang MJ, Kim Y, Kim K, Choi S (2014). Data resource profile: the Korea National Health and Nutrition Examination Survey (KNHANES). Int J Epidemiol.

[12] Stevens PE, Levin A, Kidney Disease: Improving Global Outcomes Chronic Kidney Disease Guideline Development Work Group Members (2013). Evaluation and management of chronic kidney disease: synopsis of the kidney disease: improving global outcomes 2012 clinical practice guideline. Ann Intern Med.

[13] Moon CS, Yang HR, Nakatsuka H, Ikeda M (2016). Time trend of cadmium intake in Korea. Environ Health Prev Med.

[14] Seo JW, Kim BG, Kim YM, Kim RB, Chung JY, Lee KM (2015). Trend of blood lead, mercury, and cadmium levels in Korean population: data analysis of the Korea National Health and Nutrition Examination Survey. Environ Monit Assess.

[15] Son JY, Lee J, Paek D, Lee JT (2009). Blood levels of lead, cadmium, and mercury in the Korean population: results from the Second Korean National Human Exposure and Bio-monitoring Examination. Environ Res.

[16] Kim YA, Kim YN, Cho KD, Kim MY, Kim EJ, Baek OH (2011). Blood heavy metal concentrations of Korean adults by seafood consumption frequency: using the fourth Korea National Health and Nutrition Examination Survey (KNHANES IV), 2008. Korean J Nutr.

[17] Shin JY, Lim JH, Park SG, Lee JN, Jang M, Huh CS (2004). Influence of smoking on blood cadmium concentration in university students. J Prev Med Public Health.

[18] Brockhaus A, Freier I, Ewers U, Jermann E, Dolgner R (1983). Levels of cadmium and lead in blood in relation to smoking, sex, occupation, and other factors in an adult population of the FRG. Int Arch Occup Environ Health.

[19] Repić A, Bulat P, Antonijević B, Antunović M, Džudović J, Buha A (2020). The influence of smoking habits on cadmium and lead blood levels in the Serbian adult people. Environ Sci Pollut Res Int.

[20] Bernhard D, Rossmann A, Wick G (2005). Metals in cigarette smoke. IUBMB Life.

[21] Buser MC, Ingber SZ, Raines N, Fowler DA, Scinicariello F (2016). Urinary and blood cadmium and lead and kidney function: NHANES 2007–2012. Int J Hyg Environ Health.

[22] Tsai KF, Hsu PC, Kung CT, Lee CT, You HL, Huang WT (2021). The risk factors of blood cadmium elevation in chronic kidney disease. Int J Environ Res Public Health.

[23] Kim NH, Hyun YY, Lee KB, Chang Y, Ryu S, Oh KH (2015). Environmental heavy metal exposure and chronic kidney disease in the general population. J Korean Med Sci.

[24] Wang R, Long T, He J, Xu Y, Wei Y, Zhang Y (2022). Associations of multiple plasma metals with chronic kidney disease in patients with diabetes. Ecotoxicol Environ Saf.

[25] Byber K, Lison D, Verougstraete V, Dressel H, Hotz P (2016). Cadmium or cadmium compounds and chronic kidney disease in workers and the general population: a systematic review. Crit Rev Toxicol.

[26] Kazancioğlu R (2011). Risk factors for chronic kidney disease: an update. Kidney Int Suppl.

[27] Goldstein SL, Devarajan P (2011). Acute kidney injury in childhood: should we be worried about progression to CKD?. Pediatr Nephrol.

